# The Influence of Wire Electrical Discharge Machining Cutting Parameters on the Surface Roughness and Flexural Strength of ZrO_2_/TiN Ceramic Nanocomposites Obtained by Spark Plasma Sintering

**DOI:** 10.3390/nano9101391

**Published:** 2019-09-28

**Authors:** Anton Smirnov, Anton Seleznev, Nestor Washington Solís Pinargote, Yuri Pristinskiy, Pavel Peretyagin, José F. Bartolomé

**Affiliations:** 1 Moscow State University of Technology “STANKIN”, Vadkovsky per. 1, Moscow 127055, Russian; a.seleznev@stankin.ru (A.S.);; 2 Instituto de Ciencia de Materiales de Madrid (ICMM), Consejo Superior de Investigaciones Científicas (CSIC), C/Sor Juana Inés de la Cruz 3, 28049 Madrid, Spain

**Keywords:** ceramic nanocomposites, spark plasma sintering (SPS), mechanical properties, wire electrical discharge machining (WEDM), electrical conductivity, surface quality

## Abstract

In this work, we characterized the mechanical and electrical properties of zirconia-based ceramic nanocomposites reinforced with 30 and 40 vol. % TiN and fabricated by spark plasma sintering. In addition to their improved mechanical performance, these compositions have sufficient electrical conductivity to allow wire electrical discharge machining (WEDM). The influence of WEDM variables on the roughness and the mechanical strength of samples was analyzed after each cut. The experimental results showed that the roughness of machined surfaces can be reduced by variations in WEDM manufacturing regimes, and, consequently, a drastic drop in flexural strength of workpieces can be avoided. Furthermore, the composites with higher content and homogeneous distribution of the conductive phase exhibited better surface quality as well.

## 1. Introduction

The toughening effect induced by tetragonal to monoclinic transformation in yttria-stabilized tetragonal zirconia leads to increases in the crack propagation resistance [1]. Moreover, stabilized zirconia offers exceptional resistance to thermal shock as well as physical and chemical wear, making it an ideal material for the production of many difficult applications [2,3,4,5,6,7,8,9,10] where conventional materials often fail.

However, ceramics can contain many microstructural defects of various types, shapes and sizes. These defects are primarily created during fabrication and surface machining, and their presence is unavoidable. Moreover, the machining of ceramic parts is an expensive, difficult and time-consuming process, and diamond tools are often the only tools that can be used for these operations. In this context, electrochemical processes can act as a substitute for the conventional machining of ceramics; these include wire electrical discharge machining (WEDM), which is commonly used (especially for machining hard metals) to avoid expensive grinding and labor-intensive operations for high-quality finish machining operations of workpieces.

However, WEDM only works for electrically conductive materials. In order to be machinable by WEDM, the workpieces should have an electrical resistivity of less than 1–3 Ω·m [11,12,13]. Unfortunately, monolithic zirconia has a high electrical resistivity (>10^10^ Ω·cm) and, consequently, can only be machined using conventional machining processes.

The electrical conductivity of 3Y-TZP can be increased by the embedding of secondary electrically conductive phases (carbides, nitrides, and borides). In addition, these secondary phases can help to combat the natural brittleness of ceramics and increase their reliability. Moreover, this reinforcement is very important because machining problems can arise during electric discharge due to finish conditions, corrosion of the material and the effect of machining regimes on the surface damage [14,15,16,17,18,19]. This may lead to unintended effects such as cracking in the area between the melted part and the unaffected base material. Therefore, a critical factor in manufacturing ceramic components is to perform cost-effective manufacturing without negatively affecting the mechanical properties. Additionally, it is well known that the mechanical properties of ceramic vary depending on the grain size, which in turn depends on the sintering conditions, namely, temperature and time. For that reason, the spark plasma sintering (SPS) technique can be applied to minimize undesirable grain growth and consequently, the reduction in mechanical properties. Using this solidification technology allows for the rapid fabrication of uniform, fully dense compacts at a lower temperature than conventional sintering techniques, which should help counteract the main drawback of those conventional techniques, that is, the significant grain growth of materials obtained during the process [20,21,22,23,24,25].

The aim of this article is to study the evolution of the mechanical and electrical behavior of ZrO_2_-based ceramics containing 30 or 40 vol. % TiN. Moreover, the effect of WEDM cutting regimes on the surface quality of the machined samples, which is related to the composites’ bending strength, is evaluated.

## 2. Materials and Methods

### 2.1. Nanocomposite Preparation

For this work, commercial tetragonal zirconia (3Y-TZP, d_50_ = 260 ± 0.05 nm, Tosoh Corp., Tokyo, Japan) and TiN (d_50_ = 0.9 μm, Plasmotherm, Moscow, Russia, 99.9% purity) powders were used. Powders were wet-mixed for 24 h in a plastic container on a multidirectional mixer Turbula using isopropyl as liquid media. The suspensions were freeze-dried by means of the LabConco FreeZone2.5 freeze dry system (Kansas, MO, USA) at a shelf temperature of +21 ± 2 °C, pressure 0.02 ± 0.01 mBar and condenser temperature of −50 ± 2 °C. This technique is an efficient method to achieve homogeneous particles that are free from agglomerates of TiN inside the ZrO_2_ matrix as well as avoid a sieving step. Flat discs (50 mm diameter, 5 mm thickness) were produced using a H-HP D-25 SD spark plasma sintering (SPS, FCT Systeme GmbH, Rauenstein, Germany) furnace at 1400 °C under vacuum at 80 MPa axial pressure and 3 min dwell at the final temperature. Two compositions were used: Z30T and Z40T where the numbers show the TiN volume content.

### 2.2. XRD Characterization

X-ray measurements were carried out using an Empyrean diffractometer (PANalytical, Almelo, Netherlands) with radiation source Cu–Kα (λ = 1.5405981Å) anode supplied with 60 kV and a current of 30 mA.

### 2.3. Microstructural Characterization

Scanning electron microscopy (SEM) characterization of the microstructure of samples polished down to 1 µm and as-machined samples was carried out using a VEGA 3 LMH (Tescan, Brno, Czech Republic) microscope equipped with an energy-dispersive X-ray (EDS) detector. The density (*ρ*) of the sintered specimens was determined via the Archimedes principle in distilled water. The Vickers hardness (HV) was calculated using the indenter load 9.8 N and 10 indents of each sample. Samples with dimensions of 3.0 × 4.0 × 45 mm^3^ were used to determine the fracture toughness (*K_Ic_*) using a single edge notched beam. All tests were realized at ambient temperature using the AutoGraph AG-X (Shimadzu Corp., Kyoto, Japan) universal testing machine of 5 kN capacity with a crosshead speed of 0.5 mm/min and a span of 40 mm. This method and the formulas for calculating *K_Ic_* have been reported elsewhere [26]. The same machine and specimens with the same dimensions were used for strength (*σ_f_*) determination using the three-point bending scheme. For this test, as-cut off WEDM bars and traditionally prepared bars were used as bending test specimens. For the traditional preparation method, the sintered disc was firstly cut into prismatic shaped specimens by a diamond wheel and then polished with a 1 µm polycrystalline diamond suspension. The strength was determined according to the equation:

(1) $$\sigma_{f}=\frac{3FL}{2bh^{2}}$$

where *F* is the failure load, *L* is the span, *b* is the width and *h* is the height. The strength results were averaged over 10 specimens.

### 2.4. Determination of Electrical Properties and Wire-Electroerosion Machining (WEDM)

To determine the electrical characteristics of the studied materials, four probe methods with Keithley 6220 current source (Cleveland, OH, USA) and Keithley 2182A nanovoltmeter (Cleveland, OH, USA) were employed.

Specimens were machined by wire EDM (Seibu M500SG, Seibu Electric & Machinery Co., Koga, Japan) machine. Commercial brass wire (Osaka Brasscut A500, 0.25 mm diameter) and deionized water with a conductivity of 0.1 μS/cm were taken as WEDM tools and as a dielectric, respectively. Table 1 shows the three basic machining parameters that were chosen for analysis and optimization, while other settings remained constant. The surface roughness of the processed samples was analysed using a 3D contact profilometer Talysurf CLI 500 (Taylor Hobson, Leicester, UK).

## 3. Results and Discussion

**Figure 1:**
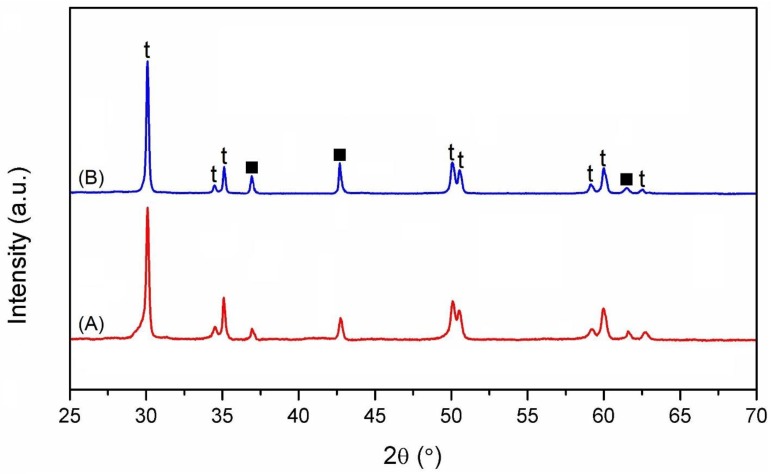
XRD patterns of polished surfaces of ZrO_2_-TiN composites with 30 (**A**) and 40 (**B**) vol. % TiN content (labelling “t” and “■” denote tetragonal zirconia and TiN, respectively).

The X-ray diffractograms of the sintered composites are shown in Figure 1, and reveal the absence of spontaneous martensitic transformation (t → m) of the zirconia particles during cooling.

Additionally, the presence of phases different to zirconia and titanium nitride was not apparent, hence, the possibility of additional compounds or contamination appearing due to processing has been discounted.

**Figure 2:**
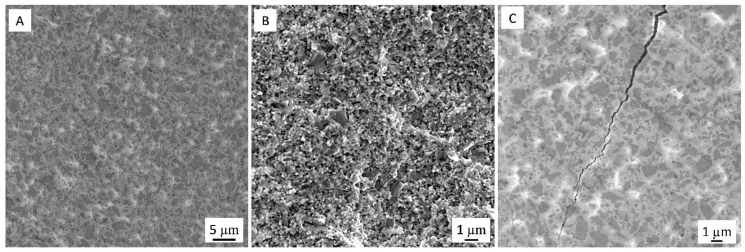
SEM micrographs of polished (**A**) and fractured (**B**) surface of zirconia-based composites with 40 vol. % TiC. (**C**) Vickers indentation crack induced on the surface of the Z40T composites. Dark phase on the micrographs is the TiN phase.

SEM images of the polished and fractured surfaces of ceramic composites are presented in Figure 2A,B.

The presence of pores was not observed in the microstructural investigation. Based on the theoretical values of density for ZrO_2_ and TiN, (6.05 and 5.43 g/cm^3^, respectively), we found that nearly fully dense (99% th.) composites were fabricated. Moreover, the powder mixing technique was appropriate, as confirmed by the uniform distribution of the second phase in the zirconia matrix.

The mechanical properties and electrical resistivity of the ceramic composites with varying TiN content were compared with the characteristics of monolithic zirconia and summarized in Table 2.

The composites exhibited a higher hardness value than monolithic zirconia, which can be attributed to the higher hardness of TiN (~ 18 GPa). The range of fracture toughness of the composites was 6–6.5 MPa·m^1/2^. Figure 2C shows the crack propagation on the polished surfaces of ZrO_2_/TiN composites produced by the Vickers indentation under a load of 9.8 N. The difference between the coefficient of thermal expansion of yttria-stabilized ZrO_2_ (α_0–1000_ °C = 12 × 10^−6^/°C) [27] and TiN (α_0–1000_ °C = 9.4 × 10^−6^/°C) [28] will cause compressive residual stresses on the TiN particles (Figure 2C) and tensile stresses on the ZrO_2_ phase that increase their transformability [26]. Both fracture toughening mechanisms can lead to a higher toughness value in the ZrO_2_/TiN composites.

The electrical conductivities of the composites were 1.08∙10^5^ and 2.26∙10^5^ S∙m^−1^ for Z30T and Z40T, respectively, which means the material is suitable for WEDM. This involves interactions between the workpiece and an electrode (gap), which creates thermoelectric energy. The unwanted material is removed from the workpiece through melting and vaporizing, when electric sparks occur between electrodes. Once the sparking cycle occurs, the plasma generates small craters in the neighborhood of the machined front. Additionally, there are regions of fused material caused by wire.

**Figure 3:**
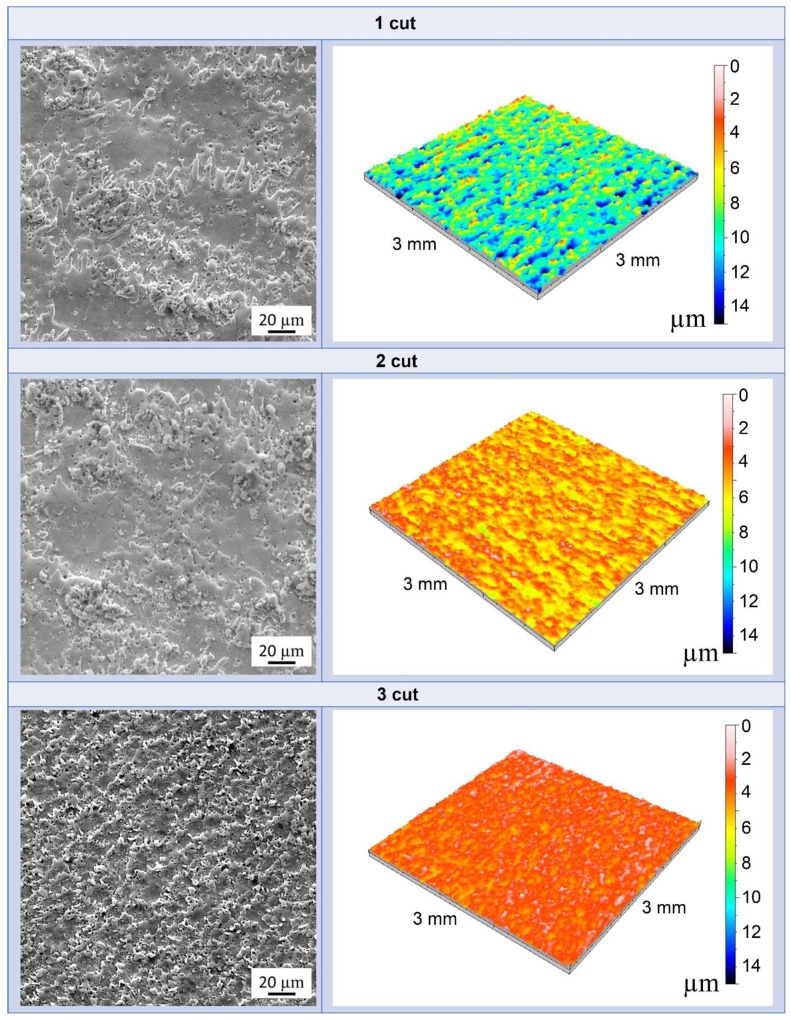
SEM images and 3D topographies corresponding to Z40T composites WEDMed surface with a depth scale (colored scale) as a function after each cut.

Figure 3 shows the SEM images and three-dimensional color topography of the WEDMed surface of Z40T composites after each cut.

The machined surfaces of both composites show splashes of re-solidified material and pores. The formation of such pores can be explained by the expansion of the nitrogen (N_2_) and hydrogen (H_2_) gases from the surface. Because water has been used as a dielectric liquid, it is likely that the formation of these gases occurs due to the oxidation of TiN to TiO_2_, as illustrated by the following equation:

(2) $$2\mathrm{TiN}+4H_{2}O\to 2{\mathrm{TiO}}_{2}+4H_{2}+N_{2}$$

**Figure 4:**
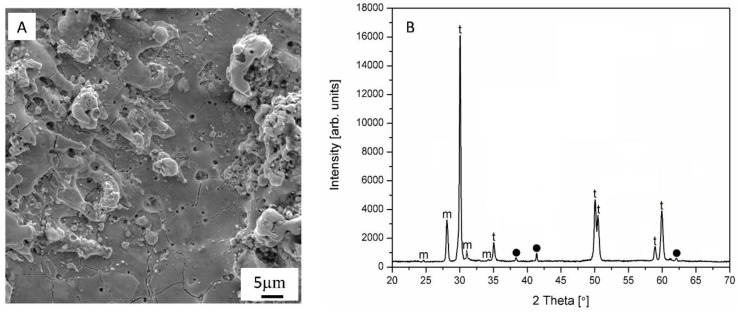
Topography (**A**) and corresponding XRD pattern (**B**) of the WEDMed surface of Z40T nanocomposite. The labels, “t” and “m” denote tetragonal and monoclinic zirconia, respectively. “●” marks TiN reflections.

The SEM image at higher magnification and the corresponding XRD spectrum image of the WEDM surface of Z40T nanocomposite after the third cut are presented in Figure 4A,B, respectively. As can be seen from the SEM image, there are many droplets left on the machined surface due to the high temperature achieved during WEDM, which contributes to material removal. The presence of these droplets suggests that the material removal mechanism is probably melting and evaporation.

Additionally, a network of cracks can be observed on the machined surface. The cracking process could be related to the field of tensile residual stresses, resulting from thermal effects due to the rapid heating and quenching cycles induced during the WEDM process. Furthermore, such cracking probably leads to the tetragonal-to-monoclinic transformation in zirconia and, consequently, to the presence of m-ZrO_2_ on the machined surface. XRD confirmed that, unlike the polished sintered samples (Figure 1), the WEDM surface layer consists not only of tetragonal and titanium nitride, but also of monoclinic zirconia (Figure 4B).

**Figure 5:**
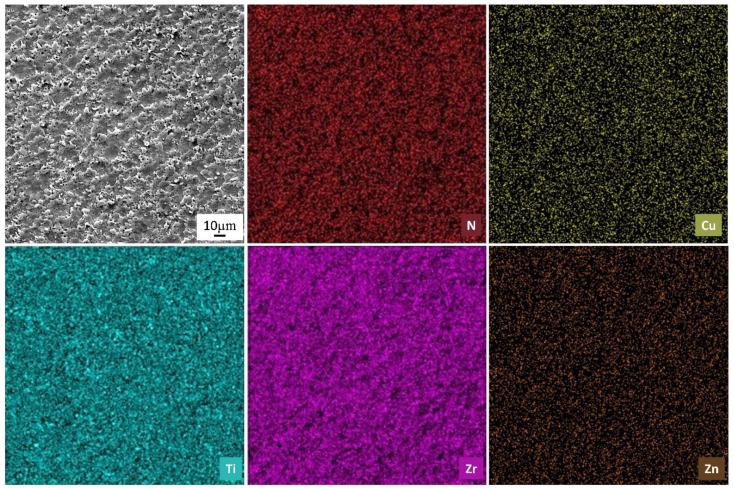
The WEDMed surface of Z40T composite and X-ray element distribution maps.

However, after the third cut a smoother surface and more homogeneous removal of material was observed. Figure 5 exhibits EDS maps of the WEDMed surface of ZrO_2_-40 vol.% TiN composites after the third cut, showing the homogeneous distribution of the elements throughout the sample.

The presence of Cu and Zn is due to the wire tool material; metal particles move from the electrode to the workpiece during machining and cannot be completely flushed out by the dielectric fluid. EDS was also used for the chemical microanalysis of the polished and machined surface after each cut. The concentration of the elements in the polished versus WEDM finished surface is given in Table 3. It is important to note that, although the EDS measurement error is high in the case of nitrogen and oxygen, the values of these elements on the polished surface are much higher (about 5 times greater) than the values corresponding to the WEDM surface, thus confirming Equation (2).

Table 4 summarizes the results obtained for the surface roughness and bending strength of WEDM specimens as a function of the machining conditions.

The surface roughness is directly related to the critical defect size on the surface of the material, and therefore to the mechanical strength value. The lower the roughness value, the higher the mechanical strength value. It is obvious that the polishing of the samples caused lower surface roughness than the wire electrical discharge machined bars. As would be expected, as a result of using this traditional method of preparation, the ZrO_2_-TiN materials have the highest bending strength: 1411 and 1423 MPa for Z30T and Z40T, respectively. However, it was revealed that the surface roughness for WEDM cut bars becomes more refined with a higher number of cuts. Therefore, the strength increases as the surface roughness decreases. In addition, the surface quality of the 40 vol. % TiN containing the composite was better after each cut compared to the 30 vol. % TiN composites due to changes in the material removal mechanism and the higher amount of secondary conductive phase.

## 4. Conclusions

The results obtained revealed that the addition of 30 and 40 vol % TiN phases to the zirconia ceramic matrix improved the mechanical performance and raised the conductivity to a level where wire electrical discharge machining is possible. Moreover, it should be noted that the electrical conductivity and surface quality were simultaneously enhanced in compositions with a higher content of TiN.

It was shown that, after WEDM, the strength is reduced due to the degradation of the surface quality of the machined ceramic nanocomposite. However, applying various cutting conditions allows for increasing the surface quality and, simultaneously, the strength of wire EDM bars to a value very close to that of the polished nanocomposites. These results show the possibility of developing novel conductive ceramic nanocomposites with enhanced mechanical properties that can be obtained by electrical discharge machining for the effective performance of many production activities. Understanding the relationship between cutting parameters, surface topology and their impact on the mechanical performance of a composite will enable the design and processing of zirconia-based ceramic parts with a complex shape.

## Figures and Tables

**Table 1:** Wire electrical discharge machining parameters.

	Machining Conditions		
	Average Working Voltage (V)	Average Working Current (A)	Off Time (μs)
1 cut	100	3.0	8
2 cut	84	2.2	4
3 cut	72	1.5	2

**Table 2:** Mechanical properties and electrical conductivity of ZrO_2_-TiN composites.

Specimen	Density [%ρ_th_] ^†^	Elastic Modulus *E* [GPa]	Hardness *HV* [GPa]	Fracture Toughness *K_Ic_* [MPa∙m^1/2^]	Electrical Conductivity × 10^5^ [S∙m^−1^]
3Y-TZP [22]	99	198 ± 5	13 ± 0.3	6.1 ± 0.3	-
Z30T	99	237 ± 7	13.9 ± 0.4	6.3 ± 0.2	1.08
Z40T	99	252 ± 8	13.8 ± 0.3	6.5 ± 0.3	2.26

^†^ Percent theoretical density.

**Table 3:** Elemental analysis of polished surface vs. WEDM surface of Z40T composite.

Element	Polished Surface		WEDM Surface					
			Cut 1		Cut 2		Cut 3	
	Wt.%	Atm.%	Wt.%	Atm.%	Wt.%	Atm.%	Wt.%	Atm.%
N	16.35	20.47	2.57	5.10	2.950	5.26	2.78	4.95
O	27.24	44.21	27.14	44.47	27.84	45.698	27.43	45.42
Ti	17.16	11.86	17.62	12.10	17.48	11.98	17.53	12.06
Y	1.21	0.49	2.03	0.62	2.14	0.82	2.09	0.78
Zr	38.04	22.97	38.58	23.72	38.17	23.47	38.36	23.58
Cu	n.a.	n.a.	4.24	3.62	3.89	3.24	4.15	3.34
Zn	n.a.	n.a.	1.65	1.15	2.08	1.44	2.02	1.09
Al	n.a.	n.a.	0.28	0.31	0.23	0.29	0.26	0.35
C	n.a.	n.a.	5.89	8.91	5.22	7.52	5.38	8.43

**Table 4:** The WEDM cutting parameters influence on surface roughness and strength values.

	Z30T				Z40T			
	Machining Conditions							
	Traditional Preparation	1 Cut	2 Cut	3 Cut	Traditional Preparation	1 Cut	2 Cut	3 Cut
Surface roughness, Sa (μm)	0.21 ± 0.02	3.08 ± 0.08	2.11 ± 0.05	1.1 ± 0.03	0.22 ± 0.02	2.84 ± 0.05	1.73 ± 0.03	0.89 ± 0.02
Flexural strength, σ_f_ (MPa)	1411 ± 32	1226 ± 102	1287 ± 61	1319 ± 37	1423 ± 29	1267 ± 87	1323 ± 43	1373 ± 31

## References

[1] Hannink R.H.J., Kelly P.M., Muddle B.C. (2000). Transformation toughening in zirconia-containing ceramics. J. Am. Ceram. Soc..

[2] Bocanegra-Bernal M.H., De La Torre S.D. (2002). Phase transitions in zirconium dioxide and related materials for high performance engineering ceramics. J. Mater. Sci..

[3] Solís N.W., Peretyagin P., Torrecillas R., Fernández A., Menéndez J.L., Mallada C., Díaz L.A., Moya J.S. (2017). Electrically conductor black zirconia ceramic by SPS using graphene oxide. J. Electroceram..

[4] Kameo K., Friedrich K., Bartolome J.F., Diaz M., Lopez-Esteban S., Moya J.S. (2003). Sliding wear of ceramics and cermets against steel. J. Eur. Ceram. Soc..

[5] Pecharroman C., Lopez-Esteban S., Bartolome J.F., Moya J.S. (2001). Evidence of nearest-neighbor ordering in wet-processed zirconia-nickel composites. J. Am. Ceram. Soc..

[6] Sergo V., Lughi V., Pezzotti G., Lucchini E., Meriani S., Muraki N., Katagiri G., Nishida T. (1998). The effect of wear on the tetragonal-to-monoclinic transformation and the residual stress distribution in zirconia-toughened alumina cutting tools. Wear.

[7] Chen Z.D., Myo M.H., Choy C.M. (2003). Rapid manufacturing of Y-TZP ceramic punch using powder injection moulding technology. Mater. Sci. Forum.

[8] Bartolome J.F., Montero I., Diaz M., Lopez-Esteban S., Moya J.S. (2004). Accelerated aging in 3-mol%-yttria-stabilized tetragonal zirconia ceramics sintered in reducing conditions. J. Am. Ceram. Soc..

[9] McEntire B.J., Bal B.S., Rahaman M.N., Chevalier J., Pezzotti G. (2015). Ceramics and ceramic coatings in orthopaedics. J. Eur. Ceram. Soc..

[10] Moya J.S., Sanchez-Herencia J.A., Bartolome J.F., Tanimoto T. (1997). Elastic modulus in rigid Al_2_O_3_/ZrO_2_ ceramic laminates. Scr. Mater..

[11] Rodriguez-Suarez T., Bartolomé J.F., Smirnov A., Lopez-Esteban S., Díaz L.A., Torrecillas R., Moya J.S. (2011). Electroconductive Alumina-TiC-Ni nanocomposites obtained by Spark Plasma Sintering. Ceram. Int..

[12] Grigoriev S., Peretyagin P., Smirnov A., Solis W., Diaz L.A., Fernandez A., Torrecillas R. (2017). Effect of graphene addition on the mechanical and electrical properties of Al_2_O_3_-SiCw ceramics. J. Eur. Ceram. Soc..

[13] Grigoriev S., Volosova M., Peretyagin P., Seleznev A., Okunkova A., Smirnov A. (2018). The Effect of TiC additive on mechanical and electrical properties of Al_2_O_3_ ceramic. Appl. Sci..

[14] Puertas I., Luis C.J., Álvarez L. (2004). Analysis of the influence of EDM parameters on surface quality, MRR and EW of WC–Co. J. Mater. Process. Technol..

[15] Khan A.A., Ali M., Shaffiar M. (2006). Relationship of surface roughness with current and voltage during wire EDM. J. Appl. Sci..

[16] Díaz L.A., Solís N.W., Peretyagin P., Fernández A., Morales M., Pecharromán C., Moya J.S., Torrecillas R. (2016). Spark plasma sintered Si_3_N_4_/TiN nanocomposites obtained by a colloidal processing route. J. Nanomater..

[17] Obara H., Satou H., Hatano M. (2004). Fundamental study on corrosion of cemented carbide during wire EDM. J. Mater. Process. Technol..

[18] Smirnov A., Peretyagin P., Bartolome J.F. (2018). Wire electrical discharge machining of 3Y-TZP/Ta ceramic-metal composites. J. Alloys Compd..

[19] Smirnov A., Peretyagin P., Bartolome J.F. (2019). Processing and mechanical properties of new hierarchical metal-graphene flakes reinforced ceramic matrix composites. J. Eur. Ceram. Soc..

[20] Rice R.W. (1997). Effects of environment and temperature on ceramic tensile strength–grain size relations. J. Mater. Sci..

[21] Eichler J., Rödel J., Eisele U., Hoffman M. (2007). Effect of grain size on mechanical properties of submicrometer 3Y-TZP: Fracture strength and hydrothermal degradation. J. Am. Ceram. Soc..

[22] Smirnov A., Kurland H.-D., Grabow J., Müller F.A., Bartolomé J.F. (2015). Microstructure, mechanical properties and low temperature degradation resistance of 2Y-TZP ceramic materials derived from nanopowders prepared by laser vaporization. J. Eur. Ceram. Soc..

[23] Viswanathan V., Laha T., Balani K., Agarwal A., Seal S. (2006). Challenges and advances in nanocomposite processing techniques. Mater. Sci. Eng. Rep..

[24] Smirnov A., Beltrán J.I., Rodriguez-Suarez T., Pecharromán C., Muñoz M.C., Moya J.S., Bartolomé J.F. (2017). Unprecedented simultaneous enhancement in flaw tolerance and fatigue resistance of zirconia–Ta composites. Sci. Rep..

[25] Smirnov A., Peretyagin P., Solís Pinargote N.W., Gershman I., Bartolomé J.F. (2019). Wear behavior of graphene-reinforced alumina–silicon carbide whisker nanocomposite. Nanomaterials.

[26] Smirnov A., Bartolomé J.F., Kurland H.-D., Grabow J., Müller F.A. (2016). Design of a new zirconia-alumina-Ta micro-nanocomposite with unique mechanical properties. J. Am. Ceram. Soc..

[27] Morrell R. (1985). Handbook of Properties of Technical & Engineering Ceramics, an Introduction for Engineer and Designer, Part 1.

[28] Pierson H.O. (1996). Handbook of Refractory Carbides and Nitrides: Properties, Characteristics, Processing and Applications.

